# A Bayesian multivariate latent *t*-regression model for assessing the association between corticosteroid and cranial radiation exposures and cardiometabolic complications in survivors of childhood acute lymphoblastic leukemia: a PETALE study

**DOI:** 10.1186/s12874-019-0725-9

**Published:** 2019-05-14

**Authors:** Miguel Caubet Fernandez, Simon Drouin, Mariia Samoilenko, Sophia Morel, Maja Krajinovic, Caroline Laverdière, Daniel Sinnett, Emile Levy, Valérie Marcil, Geneviève Lefebvre

**Affiliations:** 1 Department of Mathematics, University of Quebec at Montreal (UQAM), 201 President-Kennedy Av., Montréal, QC H2X 3Y7 Canada; 2 Division of Hematology-Oncology, Research Center, Sainte-Justine University Health Center, 3175 Chemin de la Côte-Sainte-Catherine, Montréal, QC H3T 1C5 Canada; 3 Department of Pediatrics, Faculty of Medicine, University of Montreal, Montréal, QC H3T 1C5 Canada; 4 Faculty of Pharmacy, University of Montreal, Montréal, QC H3T 1J4 Canada; 5 Research Center, Centre hospitalier de l’Université de Montréal, Montréal, Canada

**Keywords:** Childhood acute lymphoblastic leukemia, Corticosteroids, Cranial radiotherapy, Obesity, Insulin resistance, Hypertension, Dyslipidemia, Multivariate binary outcome model

## Abstract

**Background:**

Childhood acute lymphoblastic leukemia (cALL) is the most frequent pediatric cancer. Over the past decades, treatment of cALL has significantly improved, with cure rates close to 90%. However intensive chemotherapy and cranial radiotherapy (CRT) during a critical period of a child’s development have been shown to lead to significant long-term side effects including cardiometabolic complications. Using the PETALE (*Prévenir les effets tardifs des traitements de la leucémie aiguë lymphoblastique chez l’enfant*) cALL survivor cohort, we investigated the association between combined cumulative corticosteroids (CS) doses and CRT exposures and obesity, insulin resistance, (pre-)hypertension, and dyslipidemia jointly.

**Methods:**

A Bayesian multivariate latent-*t* model which accounted for our correlated binary outcomes was used for the analyses (*n* = 241 survivors). CS doses were categorized as low (LD) or high (HD). Combined exposure levels investigated were: 1) LD/no CRT; 2) LD/CRT, and; 3) HD/CRT. We also performed complementary sensitivity analyses for covariate adjustment.

**Results:**

Prevalence of cardiometabolic complications ranged from 12.0% for (pre-)hypertension to 40.2% for dyslipidemia. The fully adjusted odds ratio (OR) for dyslipidemia associated with LD/CRT (vs. LD/No CRT) was OR = 1.98 (95% credible interval (CrI): 1.02 to 3.88). LD/CRT level also led to a 0.15 (95% CrI: 0.00 to 0.29) excess risk to develop at least one cardiometabolic complication. Except for obesity, adjusted results for the highest exposure category HD/CRT were generally similar to those for LD/CRT albeit not statistically significant. White blood cell count at diagnosis, a proxy for cALL burden at diagnosis, was found associated with insulin resistance (OR = 1.08 for a 10-unit increase (× 10^9^/L), 95% CrI: 1.02 to 1.14).

**Conclusions:**

Our results indicated that combined LD/CRT exposure is a likely determinant of dyslipidemia among cALL survivors. No evidence was found to suggest that high doses of CS lead to additional risk for obesity, insulin resistance, (pre-)hypertension, and dyslipidemia beyond that induced by CRT. The multivariate model selected for analyses was judged globally useful to assess potential exposure-related concomitance of binary outcomes.

**Electronic supplementary material:**

The online version of this article (10.1186/s12874-019-0725-9) contains supplementary material, which is available to authorized users.

## Background

Childhood acute lymphoblastic leukemia (cALL) is the most frequent pediatric cancer. Over the years, treatment of cALL has significantly improved such that nearly 90% of patients are cured with current therapy regimens. This success is largely due to the progressive intensification of cALL treatment and the implementation of risk-adapted protocols [1, 2]. Despite this remarkable improvement, intensive chemotherapy and cranial radiotherapy (CRT) during a vulnerable period of a child’s development can lead to significant long-term side effects. Childhood ALL survivors are known to be at higher risk of developing late treatment-associated morbidities (late adverse effects; LAEs), with a cumulative incidence of chronic health conditions exceeding 60% [3, 4]. The PETALE (*Prévenir les effets tardifs des traitements de la leucémie aiguë lymphoblastique chez l’enfant*) study, recently conducted at Sainte-Justine University Health Center (SJUHC), was designed to identify and characterize the most common LAEs observed in cALL survivors [5].

Levy et al. [6] investigated the prevalence of cardiometabolic complications in the PETALE cohort. A total of 88 patients (35.6%) presented a single cardiometabolic outcome among obesity, (pre)-hypertension, prediabetes (insulin resistance), and dyslipidemia, while 61 patients (24.7%) cumulated two or more. A large proportion of PETALE cALL survivors received CRT, specifically those at high risk of relapse. In multiple regression analyses, CRT was found to increase the prevalence of dyslipidemia (relative risk (RR): 1.60; 95% confidence interval (CI): 1.07 to 2.41) and high low-density lipoprotein (LDL)-cholesterol (RR: 4.78; 95% CI: 1.72 to 13.28). Moreover, male gender was found positively associated with (pre)-hypertension (RR: 5.12; 95% CI: 1.81 to 14.5). When compared to the general Canadian population, Levy et al. [6] found that cALL survivors were at higher risk of having the metabolic syndrome, dyslipidemia, (pre-)hypertension and high LDL-cholesterol. However, after stratification, only CRT recipients were found more likely than healthy controls to have cardiometabolic complications.

Building upon Levy et al. [6], we performed additional analyses to shed further light on the impact of cALL treatment (exposure) received on cardiometabolic health in the PETALE cohort of survivors. Chemotherapy dosage was not evaluated in Levy et al. [6], however both radiotherapy and chemotherapy treatments may contribute to the development of cardiometabolic complications through several mechanisms, for example by damaging endocrine organs, inducing endothelial dysfunction and perturbing adipose tissue metabolism [6–8]. cALL chemotherapy includes multiple agents that are believed to be highly deleterious for cardiometabolic health, such as corticosteroids (CS). We thus investigated the association between doses of CRT and CS received and cardiometabolic complications (obesity, insulin resistance, (pre-)hypertension, and dyslipidemia). Rather than performing analyses for each cardiometabolic outcome separately, our analyses considered all outcomes simultaneously to account for likely co-occurrence between them.

Multivariate statistical models are indicated when analyzing interrelated outcome variables. Grounded in the Bayesian framework, the multivariate logistic regression model of O’Brien and Dunson [9] is an appealing model to study the association between a set of covariates and several correlated binary outcomes. A distinctive feature of this model is its ability to output results with high interpretability, by allowing for the familiar log odds ratio interpretation of regression coefficients as in a standard univariate logistic model, while accounting for outcome dependencies in an unrestricted fashion [9]. Inference based on O’Brien and Dunson’s model was recently further pushed forward by Hund et al. [10] who estimated average treatment effects for types of uranium exposure on kidney disease, hypertension, and diabetes, jointly, in the Navajo Nation. Despite its attractiveness, this Bayesian multivariate model, combined with the post-hoc analysis approach of Hund et al. [10], has not received broad attention in clinical epidemiology, perhaps due to a greater complexity. In addition to the substantive objective described above, a methodological objective was therefore to detail and emphasize the application of this well thought multivariate model on our cALL survivorship data.

In this study, we used the O’Brien and Dunson / Hund et al. global modeling approach to identify combined CS and CRT treatment levels associated with cardiometabolic LAEs in cALL survivors. The insight gained on treatment-LAE associations will further guide risk-adapted follow-up of cALL survivors. From a methodological perspective, our modeling proposal (with associated computer code) will expand health practitioners’ toolkit for the analysis of correlated binary outcome variables from a Bayesian perspective.

## Methods

### PETALE cohort

Subjects enrolled in the PETALE study were treated for cALL at SJUHC with the Dana Farber Cancer Institute (DFCI) protocols 1987–01 to 2005–01 [11, 12]. cALL survivors less than 19 years old at diagnosis, more than 5 years post diagnosis and who had never experienced a relapse were invited to participate. Between 2012 and 2016, a total of 247 participants underwent thorough clinical, biological, and psychosocial evaluations (mean age at interview: 21.6 years; standard deviation (SD): 6.3). A detailed presentation of the PETALE study and cohort is provided in Marcoux et al. [5]

### Outcome, treatment and adjustment variables

Obesity was determined by presenting at least one of two factors at interview: obese according to body mass index (BMI) or high waist circumference [13, 14]. Insulin resistance was defined by high blood fasting glucose, high glycated hemoglobin, high homeostasis model assessment (HOMA-IR) [insulin (mIU/L) x glucose (mmol/L)/22.5] values or taking medication for diabetes [15, 16]. (Pre-)hypertension was assessed according to current guidelines for arterial pressures or by being on antihypertensive medication [17, 18]. Dyslipidemia was determined by high LDL-cholesterol, high triglycerides, low high-density lipoprotein (HDL)-cholesterol, or lipid-lowering medication [19, 20]. Age- and gender-dependent cut-off values defining cardiometabolic outcomes are presented in Additional file 1: Table S1.

Cumulative doses of CS were calculated as the sum of prednisone-equivalent, body-surface area-normalized (mg/m^2^) dexamethasone, methylprednisolone, prednisolone, and prednisone doses received during the three phases of the chemotherapy treatment (induction, intensification, and consolidation). A third quartile split (using a 13,414 mg/m^2^ threshold) was used to categorize cALL survivors as having received lower (LD) or higher (HD) doses of CS. CRT exposure was defined as a binary variable (yes/no) as most survivors who received CRT had an 18 Gray (Gy) cumulative dose, with none receiving over 20 Gy. Unlike Hund et al. [10] who focused on the effect of a single binary exposure variable entered in the multivariate model each time, a joint CS/CRT exposure variable was defined using three categories: 1) LD/No CRT; 2) LD/CRT, and; 3) HD/CRT. No cALL survivors received higher doses of CS but no CRT; as such we did not consider the “HD/No CRT” category. Information concerning the three exposure categories examined was available for 241 cALL survivors (five survivors were excluded because of no information on CS and one was excluded because they were not exclusively treated by DFCI cALL protocols 1987–01 to 2005–01).

The adjustment variables considered in our analyses were sex, age at diagnosis, white blood cell (WBC) count at diagnosis, and time since diagnosis. These adjustment variables are known to be associated with cALL treatment received, the studied cardiometabolic complications, or both. Notably, age and WBC count were used as criteria to stratify patients as having standard or high risk of relapse in DFCI cALL treatment protocols 1987–01 to 2005–01 [11, 12]. WBC count was entered as a main effect term after preliminary investigations assessing other possible functional forms with one degree of freedom (e.g., log, square root). Indeed, for the sake of simplicity, and because the proposed multivariate model has a marginal logistic interpretation, we initially considered a number of multiple logistic regressions for modeling insulin resistance versus different functions of WBC. We then selected the functional form for WBC that yielded the smallest Akaike information criterion (AIC) value for the corresponding fully adjusted model.

### Multivariate model

Let ${\boldsymbol{Y}}_i=\left({Y}_i^O,{Y}_i^I,{Y}_i^H,{Y}_i^D\right)$ be the vector of binary (1/0) outcomes for the *i* th survivor, where *O*, *I*, *H*, *D* indexes obesity, insulin resistance, (pre-)hypertension, and dyslipidemia, respectively. Let ***T*** be a *p*-dimensional vector following a multivariate Student-*t* distribution with *ν* degrees of freedom, location vector ***μ*** and scale matrix **Σ** with density

$$ {St}_p\left(\boldsymbol{t}\left|\boldsymbol{\mu}, \sum, v\right.\right)=\frac{\Gamma \left(\left(v+p\right)/2\right)}{\Gamma \left(v/2\right){\left( v\pi \right)}^{p/2}\left|\mathbf{\sum}\left|{}^{1/2}\right.\right.}\times {\left(1+\frac{1}{v}{\left(\boldsymbol{t}-\boldsymbol{\mu} \right)}^{\hbox{'}}{\mathbf{\sum}}^{-1}\left(\boldsymbol{t}-\boldsymbol{\mu} \right)\right)}^{-\frac{p+v}{2}}. $$

We defined ${\boldsymbol{Z}}_i=\left({Z}_i^O,{Z}_i^I,{Z}_i^H,{Z}_i^D\right)$ as a multivariate Student-*t* vector of latent (unobserved) variables with mean ${\boldsymbol{\mu}}_i=\left({\mu}_i^O,{\mu}_i^I,{\mu}_i^H,{\mu}_i^D\right)$:

1 $$ {\mu}_i^j={\beta}_0^j+{\beta}_1^j{T}_{1,i}+{\beta}_2^j{T}_{2,i}+{\boldsymbol{\beta}}_C^j{\boldsymbol{C}}_i^{\hbox{'}},\kern1em j\in \left\{ 0,I,H,D\right\}, $$

where ***β***^*j*^ is the vector of unknown regression coefficients associated to outcome *j*, *T*_1_ and *T*_2_ are dummies indexing the 2nd and 3rd categories of treatment, respectively, as opposed to the baseline (*T*_0_: LD/no CRT), and ***C*** is the vector of adjustment covariates. The scale matrix was defined as **Σ** = *δ*^2^***R***, where ***R*** is an unstructured correlation matrix, *δ*^2^ = *π*^2^(*ν* − 2)/(3*ν*), and *ν* = 7.3. The latent variables can be viewed as underlying propensities to experience the binary cardiometabolic complications; they were linked to the outcomes by ${Y}_i^j=\mathbb{1}\left({Z}_i^j>0\right)$, where $\mathbb{1}\left(\bullet \right)$ is the indicator function. Let ***X***_*i*_ = (*T*_1,*i*_, *T*_2,*i*_, ***C***_*i*_), then the likelihood function was written as

$$ P\left({\boldsymbol{Y}}_i={\boldsymbol{y}}_i|{\boldsymbol{X}}_i,\boldsymbol{\beta}, \boldsymbol{R}\right)={\int}_{-\infty}^{\infty}\dots {\int}_{-\infty}^{\infty}\left({\prod}_j\mathbb{1}{\left({z}_i^j>0\right)}^{y_i^j}\mathbb{1}{\left({z}_i^j\le 0\right)}^{1-{y}_i^j}\right)\times {St}_4\left({\boldsymbol{z}}_i|{\boldsymbol{\mu}}_i,{\delta}^2\boldsymbol{R},\nu \right)d{\boldsymbol{z}}_i. $$

As detailed in O’Brien and Dunson [9] and further discussed in Hund et al. [10], this latent multivariate Student-*t* model with *δ*^2^ = *π*^2^(*ν* − 2)/(3*ν*) and *ν* = 7.3 has a very close resemblance with a latent multivariate logistic model with the same location vector ***μ***_*i*_ [9]. As such, each non-intercept ${\beta}_{\bullet}^j$ in (1) can be interpreted as a conditional log odds ratio. There is a specific computational advantage of working with the Student-*t* model rather than the logistic model representation. Indeed, after assigning priors to the parameters ***β*** = (***β***^*O*^, ***β***^*I*^, ***β***^*H*^, ***β***^*D*^) and ***R***, a relatively simple data-augmented Markov chain Monte Carlo (MCMC) algorithm for sampling from these parameters’ posterior distribution can be devised. This algorithm samples from the full conditional distributions of ***β*** and ***Z***_*i*_, and updates ***R*** using a Metropolis step.

### Analyses

The prior distribution was specified by *π*(***β***, ***R***) = *π*(***β***)*π*(***R***), where *π*(***R***) is uniform on the space of correlation matrices and *π*(***β***) is multivariate normal. More precisely, $\pi \left(\boldsymbol{\beta} \right)={\prod}_jN\left({\boldsymbol{\beta}}^j|{\boldsymbol{\beta}}_{\ast}^j,{\boldsymbol{\Sigma}}_{\ast}^j\right)$, where ${\boldsymbol{\beta}}_{\ast}^j=\mathbf{0}$ and ${\boldsymbol{\Sigma}}_{\ast}^j=\mathit{\operatorname{diag}}\left(1000,4,\dots, 4\right)$ for *j* ∈ {*O*, *I*, *H*, *D*}. Our choice of prior is very similar to a multivariate normal prior distribution proposed by O’Brien and Dunson [9]. For the non-intercept coefficients, a standard deviation of 2 (variance 4) implies that the bulk of the prior is between +/− 1.96*2 for the log odds ratio. We used a larger prior variance (1000 instead of 4) for the intercept coefficient ${\beta}_0^j$, *j* ∈ {*O*, *I*, *H*, *D*}, so to only weakly inform on the log odds for outcome *j* at the baseline level. Crude and adjusted analyses were performed. Because WBC count at diagnosis was not taken into account in Levy et al. [6], the sensitivity of the results to the inclusion of this covariable in the model was assessed.

We used a multivariate normal as the proposal distribution in the Metropolis step for ***R***. The parameters of this distribution were selected towards the goal to have adequate acceptance rates (near 25%). For each of the three models, we obtained 1,050,000 draws from the posterior distribution of (***β***, ***R***) and discarded the first 50,000 as burn-in. The running time was about 30 min for fitting the most complex model on an Intel core i7 3930k 3.2GHz with 32.0 GB RAM. For each model, we reduced the autocorrelation between the successive draws for the elements of ***R*** by applying a thinning with a factor of 100, leaving a total of 10,000 draws available for inference. Chain convergence was assessed visually for every parameter using ten sequential boxplots of 1000 draws each. No convergence problems were further detected according to the Geweke diagnostic test [21]. For the full model, the effective sample size for each of the 28 regression coefficients (***β***) was 10,000 according to the R package *coda* function *effectiveSize* [22]. The effective sample sizes for the 6 unique elements of the correlation matrix ***R*** ranged between 4317 and 5325.

Standard exposure association measures were first obtained for each outcome separately for each model considered and every retained posterior draw for (***β***, ***R***). Specifically, in addition to conditional odds ratios (ORs), conditional risk differences were computed for each survivor under both levels of treatment compared (*T*_*l*_ vs. *T*_0_, *l* = 1, 2) and subsequently averaged to produce population risk differences (PRDs). The multivariate model also enabled the calculation of the difference in probability of a given configuration of the outcome vector under both levels of treatment (*T*_*l*_ vs. *T*_0_):

2 $$ {\displaystyle \begin{array}{c}{P}^{T_l}\left({Y}_i^O={y}_i^O,{Y}_i^I={y}_i^I,{Y}_i^H={y}_i^H,{Y}_i^D={y}_i^D\left|{\boldsymbol{C}}_i\right.\right)\\ {}-{P}^{T_0}\left({Y}_i^O={y}_i^O,{Y}_i^I={y}_i^I,{Y}_i^H={y}_i^H,{Y}_i^D={y}_i^D\left|{\boldsymbol{C}}_i\right.\right),\end{array}} $$

for ${y}_i^j\in \left\{0,1\right\}$, where, for example,

$$ {P}^{T_1}\left({Y}_i^O=1,{Y}_i^I=0,{Y}_i^H=0,{Y}_i^D=1|{\boldsymbol{C}}_i\right) $$

$$ ={\int}_0^{\infty }{\int}_{-\infty}^0{\int}_{-\infty}^0{\int}_0^{\infty }{St}_4\left({\boldsymbol{z}}_i|{\boldsymbol{\mu}}_i^{T_1},{\delta}^2\boldsymbol{R},\nu\ \right)d{z}_i^Od{z}_i^Id{z}_i^Hd{z}_i^D, $$

with ${\boldsymbol{\mu}}_i^{T_1}=\left({\mu}_i^{O,{T}_1},{\mu}_i^{I,{T}_1},{\mu}_i^{H,{T}_1},{\mu}_i^{D,{T}_1}\right)$ and ${\mu}_i^{j,{T}_1}={\beta}_0^j+{\beta}_1^j\cdot 1+{\beta}_2^j\cdot 0+{\boldsymbol{\beta}}_C^j{\boldsymbol{C}}_i^{\hbox{'}}$. For each of the 2^4^ = 16 outcome configurations, (2) was obtained using the R function *pmvt* which evaluated the latent variables’ multivariate Student-*t* cumulative distribution function for limits of integration compatible with the configuration. Each difference (2) was then averaged across individuals to obtain a population value, for example,

$$ {P}^{T_1}\left({Y}_i^O=1,{Y}_i^I=0,{Y}_i^H=0,{Y}_i^H=1\right)-{P}^{T_0}\left({Y}_i^O=1,{Y}_i^I=0,{Y}_i^H=0,{Y}_i^H=1\right)= $$

$$ \frac{1}{n}\sum \limits_{i=1}^n\left\{\ {P}^{T_1}\left({Y}_i^O=1,{Y}_i^I=0,{Y}_i^H=0,{Y}_i^D=1|{\boldsymbol{C}}_i\right)-{P}^{T_0}\left({Y}_i^O=1,{Y}_i^I=0,{Y}_i^H=0,{Y}_i^D=1|{\boldsymbol{C}}_i\right)\right\}. $$

As in Hund et al. [10], contrasts in probabilities for the presence of multiple cardiometabolic outcomes (*P*(*N* = ∑_*j*_*Y*^*j*^ ≥ 1, 2, 3, 4)) given treatment levels compared were also obtained. For example, the population risk difference for at least three cumulative cardiometabolic outcomes for LD/CRT versus LD/no CRT (${P}^{T_1}\left(N\ge 3\right)-{P}^{T_0}\left(N\ge 3\right)$) was calculated as

$$ \left\{{P}^{T_1}\left({Y}_i^O=1,{Y}_i^I=1,{Y}_i^H=1,{Y}_i^D=1\right)-{P}^{T_0}\left({Y}_i^O=1,{Y}_i^I=1,{Y}_i^H=1,{Y}_i^{\mathrm{D}}=1\right)\right\}+ $$

$$ \left\{{P}^{T_1}\left({Y}_i^O=1,{Y}_i^I=1,{Y}_i^H=1,{Y}_i^D=0\right)-{P}^{T_0}\left({Y}_i^O=1,{Y}_i^I=1,{Y}_i^H=1,{Y}_i^D=0\right)\right\}+ $$

$$ \left\{{P}^{T_1}\left({Y}_i^O=1,{Y}_i^I=1,{Y}_i^H=0,{Y}_i^D=1\right)-{P}^{T_0}\left({Y}_i^O=1,{Y}_i^I=1,{Y}_i^H=0,{Y}_i^D=1\right)\right\}+ $$

$$ \left\{{P}^{T_1}\left({Y}_i^O=1,{Y}_i^I=0,{Y}_i^H=1,{Y}_i^D=1\right)-{P}^{T_0}\left({Y}_i^O=1,{Y}_i^I=0,{Y}_i^H=1,{Y}_i^D=1\right)\right\}+ $$

$$ \left\{{P}^{T_1}\left({Y}_i^O=0,{Y}_i^I=1,{Y}_i^H=1,{Y}_i^D=1\right)-{P}^{T_0}\left({Y}_i^O=0,{Y}_i^I=1,{Y}_i^H=1,{Y}_i^D=1\right)\right\}. $$

We obtained draws from the posterior distributions of target association measures and calculated corresponding posterior means and equal-tailed 95% credible intervals (CrI). Because the posterior distributions of ORs were skewed, posterior medians were presented and favored for this association measure. Due to higher computational complexity, cumulative PRDs were computed on the supercomputer Briarée maintained by Calcul Québec.

## Results

Descriptive statistics on the 241 PETALE cALL survivors included in our analyses are presented in Table 1. Males were approximately as represented as females (118/241 or 49.0%), the mean age at cALL diagnosis was 6.6 years (SD = 4.6), and the mean time elapsed between diagnosis and interview was 15.4 years (SD = 5.1). A total of 99 (41.1%) cALL survivors received treatment level LD/No CRT, 82 (34.0%) received LD/CRT, and 60 (24.9%) HD/CRT. The prevalence of the four cardiometabolic complications at interview was relatively high, ranging from 12.0% for (pre-) hypertension to 40.2% for dyslipidemia. On average, cALL survivors who received the baseline level of treatment (LD/No CRT) were the youngest at diagnosis, had the lowest WBC count at diagnosis, and included slightly more females compared to the other two groups of exposure (LD/CRT and HD/CRT). Imbalance in the distribution of time since diagnosis was also observed across levels of treatment, with longer times associated with the highest exposure category (HD/CRT).

**Table 1:** Characteristics of the cALL survivor cohort

	Full cohort	LD/No CRT	LD/CRT	HD/CRT
	(*n* = 241)	(*n* = 99)	(*n* = 82)	(*n* = 60)
Obesity	76 (31.5%)	27 (27.3%)	30 (36.6%)	19 (31.7%)
Insulin resistance	41 (17.0%)	13 (13.1%)	15 (18.3%)	13 (21.7%)
(Pre-)hypertension	29 (12.0%)	8 (8.08%)	12 (14.6%)	9 (15.0%)
Dyslipidemia	97 (40.2%)	30 (30.3%)	37 (45.1%)	30 (50.0%)
Gender (male)	118 (49.0%)	41 (41.4%)	45 (54.9%)	32 (53.3%)
Corticosteroid dose (mg/m^2^)	11,192 (5169)	7603 (1322)	9747 (2480)	19,087 (3071)
Age at diagnosis (years)	6.59 (4.59)	5.30 (3.43)	7.91 (5.18)	6.91 (4.89)
Time since diagnosis (years)	15.4 (5.10)	14.0 (4.74)	14.4 (5.42)	18.9 (3.33)
WBC count at diagnosis (10^9^/L)	30.2 (53.4)	8.28 (7.84)	40.5 (58.8)	52.2 (72.7)

Results are presented as frequency and % (in parenthesis) for the binary variables and as mean and standard deviation (in parenthesis) for the continuous variables. *LD* low dose corticosteroids, *HD* high dose corticosteroids, *CRT* cranial radiotherapy, *WBC* white blood cell

Crude and adjusted results pertaining to treatment are presented individually for each outcome in Tables 2 and 3. The median-based fully adjusted OR for dyslipidemia associated with treatment LD/CRT (as opposed to LD/No CRT) was OR = 1.98 (95% CrI: 1.02 to 3.88) and the corresponding risk difference was PRD = 0.15 (95% CrI: 0.00 to 0.29). The dyslipidemia OR and PDR for HD/CRT (as opposed to LD/No CRT) were similar to those obtained for LD/CRT but they were not statistically significant, likely due to a smaller sample size in that combined exposure category (*n* = 82 versus *n* = 60). All other fully adjusted ORs and PRDs were not significantly different from their reference values (one and zero, respectively). Association measures were globally sensitive to the inclusion or exclusion of WBC count at diagnosis in the model, suggesting that it could be a confounder for studied treatment and outcomes. In particular, association measures related to obesity and insulin resistance were attracted towards the null when adjusting for this covariable.

**Table 2:** Crude and adjusted odds ratios (ORs) associated with treatment for individual cardiometabolic risk factors

OR^a^ (95% credible interval)^b^						
	Crude		Adjusted (including WBC count)		Adjusted (without WBC count)	
Obesity						
LD/CRT	1.64/1.56	(0.84, 2.93)	1.64/1.53	(0.77, 3.07)	1.79/1.69	(0.88, 3.32)
HD/CRT	1.29/1.21	(0.60, 2.40)	1.03/0.94	(0.40, 2.19)	1.19/1.10	(0.51, 2.37)
Insulin resistance						
LD/CRT	1.43/1.32	(0.61, 2.94)	1.28/1.16	(0.47, 2.84)	1.69/1.55	(0.68, 3.55)
HD/CRT	1.97/1.82	(0.81, 4.02)	1.22/1.08	(0.41, 2.78)	1.85/1.67	(0.69, 4.01)
(Pre-)hypertension						
LD/CRT	2.02/1.82	(0.75, 4.49)	1.90/1.67	(0.63, 4.51)	1.77/1.56	(0.61, 4.16)
HD/CRT	2.00/1.79	(0.69, 4.54)	2.25/1.84	(0.56, 6.22)	2.00/1.70	(0.54, 5.25)
Dyslipidemia						
LD/CRT	1.94/1.86	(1.02, 3.33)	2.11/1.98	(1.02, 3.88)	1.82/1.72	(0.91, 3.31)
HD/CRT	2.36/2.23	(1.16, 4.26)	1.95/1.80	(0.81, 3.99)	1.55/1.44	(0.69, 3.04)

Point estimates are posterior means/medians based on 10,000 draws. *LD* low-dose corticosteroids, *HD* high-dose corticosteroids, *CRT* cranial radiotherapy, *WBC* white blood cell. ^a^: The baseline treatment level is LD/No CRT. ^b^: Endpoints are 2.5 and 97.5 empirical percentiles

**Table 3:** Crude and adjusted population risk differences (PRDs) associated with treatment for individual cardiometabolic risk factors

	PRD^a^ (95% credible interval)^b^					
	Crude		Adjusted (including WBC count)		Adjusted (without WBC count)	
Obesity						
LD/CRT	0.10	(−0.04, 0.23)	0.09	(−0.05, 0.22)	0.11	(−0.03, 0.24)
HD/CRT	0.04	(−0.10, 0.18)	−0.01	(−0.16, 0.15)	0.02	(−0.12, 0.17)
Insulin resistance						
LD/CRT	0.04	(−0.06, 0.14)	0.02	(−0.09, 0.13)	0.06	(−0.05, 0.16)
HD/CRT	0.09	(−0.03, 0.20)	0.01	(−0.10, 0.14)	0.07	(−0.05, 0.19)
(Pre-)hypertension						
LD/CRT	0.06	(−0.03, 0.15)	0.05	(−0.04, 0.14)	0.04	(−0.05, 0.13)
HD/CRT	0.06	(−0.03, 0.16)	0.06	(−0.05, 0.19)	0.05	(−0.05, 0.17)
Dyslipidemia						
LD/CRT	0.14	(0.00, 0.27)	0.15	(0.00, 0.29)	0.12	(−0.02, 0.26)
HD/CRT	0.19	(0.03, 0.34)	0.13	(−0.05, 0.31)	0.08	(−0.08, 0.25)

Point estimates are posterior means based on 10,000 draws. *LD* low-dose corticosteroids, *HD* high-dose corticosteroids, *CRT* cranial radiotherapy, *WBC* white blood cell. ^a^: The baseline treatment level is LD/No CRT. ^b^: Endpoints are 2.5 and 97.5 empirical percentiles

Examining the elements of ***R*** in the fully adjusted model, we observed a positive residual correlation (i.e., a correlation not induced by treatment and covariates) between obesity and dyslipidemia (*ρ* = 0.36, 95% CrI: 0.16 to 0.54) and an even stronger one between obesity and insulin resistance (*ρ* = 0.69, 95% CrI: 0.51 to 0.83). Table 4 presents PRDs for accumulation of cardiometabolic complications. In the fully adjusted model, the treatment level LD/CRT led to a 0.15 increased probability (95% CrI: 0.02 to 0.27) to develop at least one cardiometabolic complication (*N* ≥ 1), as compared to the baseline treatment level (LD/No CRT). The magnitude of this excess risk was mainly driven by configurations (*Y*^*O*^ = 0, *Y*^*I*^ = 0, *Y*^*H*^ = 0, *Y*^*D*^ = 1) and (*Y*^*O*^ = 1, *Y*^*I*^ = 0, *Y*^*H*^ = 0, *Y*^*D*^ = 1) for which the posterior means for averaged differences (2) were 0.04 and 0.05, respectively. The PRDs for *N* ≥ 1, 2 were significantly different from zero in the adjusted model without WBC count, but these results should be interpreted with caution in light of the potentially confounding effect of WBC. Nonetheless, these results highlight an intrinsic advantage of multivariate modeling as opposed to standard univariate modeling. Indeed, while in that partially adjusted model exposure level LD/CRT was not found associated with any of the outcome marginally (see rightmost column of Tables 2 and 3), it was found associated with the occurrence of at least one or two metabolic complications when globally accounting for these outcomes (see rightmost column of Table 4).

**Table 4:** Population risk differences (PRDs) for cumulative metabolic outcomes (*N* = ∑_*j*_*Y*^*j*^)

	PRD^a^ (95% credible interval)^b^			
	Adjusted (including WBC count)		Adjusted (without WBC count)	
*N* ≥ 1				
LD/CRT	0.15	(0.02, 0.27)	0.14	(0.02, 0.26)
HD/CRT	0.11	(−0.05, 0.26)	0.10	(−0.05, 0.24)
*N* ≥ 2				
LD/CRT	0.11	(−0.01, 0.22)	0.12	(0.01, 0.23)
HD/CRT	0.05	(−0.08, 0.19)	0.07	(−0.05, 0.20)
*N* ≥ 3				
LD/CRT	0.04	(−0.01, 0.10)	0.05	(−0.00, 0.11)
HD/CRT	0.03	(−0.03, 0.09)	0.04	(−0.01, 0.11)
*N* = 4				
LD/CRT	0.01	(−0.00, 0.02)	0.01	(−0.00, 0.02)
HD/CRT	0.01	(−0.00, 0.02)	0.01	(−0.00, 0.02)

Point estimates are posterior means based on 10,000 draws. *LD* low-dose corticosteroids, *HD* high-dose corticosteroids, *CRT* cranial radiotherapy, *WBC* white blood cell. ^a^: The baseline treatment level is LD/No CRT. ^b^: Endpoints are 2.5 and 97.5 empirical percentiles

The ORs associated with the adjustment variables for the four metabolic outcomes across the two adjusted models considered (full, full without WBC count) are presented in Additional file 2: Table S2. We found that being male decreased the chance of being obese but increased the chance of being hypertensive. Moreover, time since diagnosis was positively associated with dyslipidemia in the models. These associations were also reported in Levy et al. [6], although therein male gender was found protective for obesity in unadjusted analysis only. Finally, WBC count at diagnosis was the only covariable associated with insulin resistance in the fully adjusted marginal model (1) (OR = 1.08 for a 10-unit increase (× 10^9^/L), 95% CrI: 1.02 to 1.14).

## Discussion

Our study aimed at providing additional insights on the treatment-associated risk of developing cardiometabolic complications among survivors of cALL. More precisely, the present analysis of 241 children, adolescents, and young adult cALL survivors of the PETALE cohort [5] was designed to inform on the association between combined exposures to CRT and CS doses and cardiometabolic LAEs. Moreover, since obesity, insulin resistance, hypertension, and dyslipidemia form a cohesive group of outcomes as part of the metabolic syndrome, the analysis was done using a multivariate modeling strategy enabling both marginal and joint risk assessments. Indeed, for each set of adjustment covariates, we assessed the impact of exposure on the outcomes marginally, jointly and cumulatively using a single model along with corresponding estimated regression parameters. A multivariate approach such as ours provides a more thorough description of which outcomes are affected by a change in the level of exposure, better controls for Type I error rate, and generally increases statistical power as compared to a series of univariate analyses [23, 24].

Although modern cALL treatment protocols are phasing out CRT due to severe toxicities [25], a large fraction of cALL survivors have been exposed to CRT. In our study, we found that cALL survivors who had received a lower cumulative dose of CS with CRT were at increased risk of presenting dyslipidemia: there were 15 excess cases of dyslipidemia per 100 survivors in the LD/CRT group, as compared to the LD/No CRT group. For survivors who had received LD/CRT, we similarly found 15 additional cases presenting one or more cardiometabolic complications per 100 compared to the baseline treatment group. Levy et al. [6] found, using the same cohort of cALL survivors, that CRT recipients had a 60% increased risk to develop dyslipidemia. Our results are thus consistent with those, although our risk difference assessment arguably provides a more clinically useful measure of treatment-related burden.

CRT has been proposed as a contributing factor to the development of cardiometabolic complications and a higher prevalence has been reported when chemotherapy was combined with radiotherapy [26]. As a single treatment exposure, CRT has previously been found associated with dyslipidemia in different cohorts of cALL survivors [8, 27, 28]. van Waas et al. observed that cALL survivors who received CRT had higher total cholesterol levels compared with those who did not, whereas their HDL-cholesterol levels did not differ [28]. Nottage et al. revealed that CRT exposure was associated with an increased risk for abnormal values of LDL-cholesterol, triglycerides, and HDL-cholesterol using St. Jude Lifetime (SJLIFE) cALL survivors treated between 1962 and 2002 and at least 10 years from diagnosis as basis [8]. The impact of CS doses on dyslipidemia development has been less studied. Nottage et al. [8] found no clinically meaningful associations between chemotherapeutic agents, including cumulative prescribed prednisone-equivalent doses, and abnormal lipid levels or other cardiometabolic complications. In our study, the OR and PRD for dyslipidemia associated with HD/CRT treatment (versus LD/No CRT) were of similar magnitude, although not significant, as those found for LD/CRT. Taken together, reported results suggest that CRT, but not CS dose, may be the key contributing factor in the development of dyslipidemia. Notwithstanding this insight, the negative impact of high doses of CS on lipid profile has been described. Co-administration of asparaginase and steroids was shown to cause significant changes in serum lipid levels during cALL treatment [29]. A similar effect due to high-dose CS was observed after organ transplantation [30]. However, these adverse events are known to be transient and, in light of both PETALE and SJLIFE studies, results so far do not support that these acute effects translate into increased dyslipidemia risk in the long-term.

While treatment exposure was not found associated with obesity in our study, CS have been implicated in modifications of the physiology of adiposity. Significant weight gain was shown during the 2 year period of maintenance chemotherapy, when patients are exposed monthly to pulses of high-dose CS [31, 32]. Decreased total lean body mass and increased percentage of fat mass have also been strongly associated with treatment combinations that included platinum, CRT, and/or steroids [7]. Some groups of authors demonstrated that glucocorticoids, including dexamethasone, increase BMI and serum leptin levels [33, 34]. However, among survivors of cALL, dexamethasone was associated with greater BMI during therapy, but was not a contributor at follow-up [35].

Chronic low-grade inflammation is believed to be a major contributing factor for insulin resistance development [36]. ALL and its intensive treatments constitute a fertile ground for the generation of oxidative stress and inflammation. Most chemotherapy agents, particularly doxorubicin, induce pro-inflammatory reactions [37] and have pro-inflammatory effects on the adipose tissue [38]. Furthermore, literature on the impact of oxidative stress on leukemogenesis and anticancer therapies is emerging [39–42]. Interestingly, our analyses suggested that CS and CRT may not be the primary determinant in the high prevalence of insulin resistance among cALL survivors. Rather, disease burden at diagnosis, as represented by WBC count at diagnosis, might be implicated in the physiopathology of the insulin resistance development. Activation of the immune system and inflammation processes may be detected by an increase in a number of markers, including WBC. Accordingly, a link between elevated WBC count and type 2 diabetes has been found in different populations [43, 44] and in a meta-analysis based on 20 studies [45]. This meta-analysis performed in 2010 also showed that higher levels of granulocytes and lymphocytes, but not monocytes, were associated with incident type 2 diabetes. On their part, Chee-Tin et al. found that all WBC subtypes were independently associated with insulin resistance [46]. Using data from the National Health and Nutrition Examination Survey Epidemiologic Follow-up Study (NHEFS), Ford reported an increasing dose-response relationship between leukocyte concentrations and diabetes incidence (hazard ratio for concentration stratum 9.1-56 × 10^9^/L versus stratum 2.1–5.7 × 10^9^/L: 1.50; 95% CI: 1.12, 1.99) [47].

In our cohort, which features a large window of WBC count outside the normal (4-11 × 10^9^/L) range (min: 0.42 × 10^9^/L; max: 361 × 10^9^/L; median: 9.60 × 10^9^/L; interquartile range: 25.6 × 10^9^/L), an increasing dose-response relationship between WBC count at diagnosis and insulin resistance was also observed. A hypothesis for that finding could be that high titers of WBC cause widespread and severe tissue infiltration, thus triggering a chronic inflammation feedback loop. This mechanism could be similar to that of type 2 diabetes, with a self-sustaining low-grade visceral inflammation loop (reviewed in Zand et al.) [48]. To our knowledge, our study is the first to have investigated and outlined a possible association between cALL burden at diagnosis through WBC count at diagnosis and cardiometabolic LAEs in cALL survivors including insulin resistance. Although further studies are needed to replicate this finding in other cALL survivor cohorts and investigate the physiological mechanisms underlying this phenomenon, WBC count at diagnosis appears to be a predictive factor of insulin resistance in cALL survivors.

## Conclusions

In this work, we took an innovative methodological approach based on a Bayesian multivariate latent-*t* model for evaluating the impact of cALL treatment on the cardiometabolic health of cALL survivors. Our sophisticated analysis of the PETALE cohort data indicated that combined cranial radiotherapy and low doses of corticosteroids were a determinant of dyslipidemia among cALL survivors and were associated with presenting one or more cardiometabolic LAEs. However, we could not similarly conclude that combined exposure to cranial radiotherapy and high doses of corticosteroids were associated with the accumulation of metabolic complications. These inconclusive results should not be interpreted as a lack of global impact of treatment as many aspects of the data may have obscured findings, such as statistical power and confounding effects due to other chemotherapy agents. Notwithstanding this, our multivariate model and its post-hoc analysis were found valuable, although computationally more intensive than standard statistical approaches based on univariate logistic or log-binomial models. In particular, the use of the R function *pmvt* which evaluated the latent variables’ multivariate Student-*t* cumulative distribution function was found to be a bottleneck for the computation of the cumulative population risk differences. How to decrease the running time for these calculations would need to be investigated. One possible mitigation could be to evaluate a cumulative risk difference for a typical individual of the population (based on mean or median covariate values, for example) instead of averaging cumulative risk differences over all individuals.

Two frequentist approaches often used for modeling correlated binary data are the logistic generalized estimation equation (GEE) approach [49] and the mixed effects logistic approach, the latter often referred to as a generalized linear mixed model (GLMM) [50, 51]. Because the GEE approach does not completely specify the joint distribution of the outcomes, it would not be possible to calculate multivariate outcome probabilities for obtaining cumulative population risk differences such as was done with proposed multivariate model. While a likelihood is specified in a logistic mixed effects model, the interpretation of the regression coefficients in this model is more difficult than in ours since it is conditional on the random effect(s), and integrating out these effects would imply a loss in the logistic structure. The multivariate logistic regression approach that we considered combines a full likelihood approach with ease of interpretation of the regression coefficients by directly enabling a marginal log odds ratio interpretation. The GEE approach relies on large sample arguments which are not necessary satisfied when dealing with samples of small or moderate sizes (such as the PETALE cohort). In comparison, the proposed multivariate logistic regression approach is exact, notwithstanding the number of MCMC iterations which can be increased with relative ease. In contrast to GLMM, this Bayesian model also yields, under mild conditions that can be easily verified, a proper posterior distribution when a noninformative prior is used [9].

Exemplified by the use of an unstructured correlation matrix ***R*** in our analysis, Agresti [52] highlighted the flexibility of the multivariate logistic distribution underlying the proposed model. Copula approaches are other alternatives to flexibly model correlated binary outcomes. e.g. [53–55]. For example, Genest et al. [53] introduced a multivariate approach with logistic regression margins combined to a meta-elliptical copula (e.g., normal or Student-*t*) to model residual dependency. Similarly to our studied model, copula approaches on discrete data are prone to estimation challenges. For their model, Genest et al. [53] proposed a composite likelihood method to alleviate numerical problems for the estimation of the copula (correlation) parameters; an explicit estimator for the asymptotic variance of the correlation parameter’s estimator via linearization was further provided. Overall, we consider the Bayesian multivariate latent-*t* model investigated as a principled and convenient tool for obtaining a thorough assessment of the possible relationship between an exposure (possibly multicategorical) and a set of correlated binary outcomes.

## Additional files

Additional file 1: **Table S1**. Cut-off values for cardiometabolic outcomes. (DOCX 25 kb)

Additional file 2: **Table S2.** Adjusted odds ratios (ORs) for individual cardiometabolic outcomes. (DOCX 18 kb)

Additional file 3: Supplementary Material: Bayesian multivariate latent-*t* model MCMC sampler. Rcpp code implementing the MCMC algorithm for sampling from the posterior distribution of the model parameters (ZIP 3 kb)

Additional file 4: Supplementary Material: Bayesian multivariate latent-*t* model implementation, R code example to simulate data and to fit the studied multivariate model by calling the MCMC algorithm function. (ZIP 2 kb)

Additional file 5: Supplementary Material: population risk differences calculation. R code to calculate marginal and cumulative population risk differences using the output of the MCMC sampler. (ZIP 2 kb)

## Abbreviations

(c)ALL: (childhood) acute lymphoblastic leukemia
AIC: Akaike information criterion
BMI: Body mass index
CI: Confidence interval
CrI: Credible interval
CRT: Cranial radiotherapy
CS: Corticosteroids
DFCI: Dana Farber Cancer Institute
GEE: Generalized estimation equation
GLMM: Generalized linear mixed model
Gy: Gray
HD: High dose
HDL: High-density lipoprotein
HOMA-IR: Homeostasis model assessment of insulin resistance
LAE: Late adverse effect
LD: Low dose
LDL: Low-density lipoprotein
MCMC: Markov chain Monte Carlo
NHEFS: National Health and Nutrition Examination Survey Epidemiologic Follow-up Study
OR: Odds ratio
PETALE: Prévenir les effets tardifs des traitements de la leucémie aiguë lymphoblastique chez l’enfant
PRD: Population risk difference
RR: Relative risk
SD: Standard deviation
SJLIFE: St. Jude Lifetime
SJUHC: Sainte-Justine University Health Center
WBC: White blood cell
