# Supplementary material for: A Bayesian multivariate latent t-regression model for assessing the association between corticosteroid and cranial radiation exposures and cardiometabolic complications in survivors of childhood acute lymphoblastic leukemia: a PETALE study
Source: BMC Med Res Methodol. 2019 May 14;19:100. doi: 10.1186/s12874-019-0725-9 (PMC6515639; doi:10.1186/s12874-019-0725-9)
Supplement: Supplementary file 1 — Table S1. Cut-off values for cardiometabolic outcomes. (DOCX 25 kb) [file 12874_2019_725_MOESM1_ESM.docx]

**Supplementary Table 1. Cut-off values for cardiometabolic outcomes.**

| **Outcome** | | **Adults** | **Children** |
| --- | --- | --- | --- |
| **Obesity** | |  |  |
|  | BMI | ≥30 kg/m^2^ | ≥97^th^ percentile^14^ |
|  | Waist circumference | men: ≥102 cm; women: ≥88 cm | ≥95^th^ percentile^13^ |
| **Insulin resistance** | |  |  |
|  | Blood fasting glucose | ≥6.1 mmol/L | ≥6.1 mmol/L |
|  | Glycated hemoglobin | ≥6% | ≥6% |
|  | HOMA-IR | ≥2.86 | ≥95^th^ percentile^15^ |
| **(Pre-)hypertension** | |  |  |
| SBP/DBP | | ≥130/85 mmHg | ≥90^th^ percentile for age and height^17^ |
| **Dyslipidemia** | |  |  |
|  | Triglycerides | ≥1.7 mmol/L^20^ | ≥1.47 mmol/L^19^ |
|  | LDL-cholesterol | ≥3.4 mmol/L^20^ | ≥3.36 mmol/L^19^ |
|  | HDL-cholesterol | men: <1.03 mmol/L; women <1.3 mmol/L^20^ | <1.03 mmol/L^19^ |

BMI, body mass index; SBP, systolic blood pressure; DBP, diastolic blood pressure; HOMA-IR, homeostasis model assessment; LDL, low-density lipoprotein; HDL, high-density lipoprotein.
