# Supplementary material for: A Bayesian multivariate latent t-regression model for assessing the association between corticosteroid and cranial radiation exposures and cardiometabolic complications in survivors of childhood acute lymphoblastic leukemia: a PETALE study
Source: BMC Med Res Methodol. 2019 May 14;19:100. doi: 10.1186/s12874-019-0725-9 (PMC6515639; doi:10.1186/s12874-019-0725-9)
Supplement: Supplementary file 2 — Table S2. Adjusted odds ratios (ORs) for individual cardiometabolic outcomes. (DOCX 18 kb) [file 12874_2019_725_MOESM2_ESM.docx]

**Supplementary Table 2: Adjusted odds ratios (ORs) for individual cardiometabolic outcomes.**

| OR (95% credible interval) | | | | | |  |
| --- | --- | --- | --- | --- | --- | --- |
|  | Adjusted  (including WBC count) | | Adjusted  (without WBC count) | | |  |
| **Obesity** | | | | | | |
| LD/CRT^a^ | 1.636/1.533 | (0.768, 3.073) | | 1.793/1.688 | (0.878, 3.324) |  |
| HD/CRT^a^ | 1.031/0.939 | (0.403, 2.192) | | 1.188/1.099 | (0.508, 2.369) |  |
| Age at diag. (yrs) | 1.027/1.027 | (0.960, 1.097) | | 1.024/1.023 | (0.959, 1.092) |  |
| Time since diag. (yrs) | 1.043/1.042 | (0.980, 1.108) | | 1.037/1.037 | (0.974, 1.103) |  |
| Male | 0.415/0.397 | (0.224, 0.707)* | | 0.420/0.402 | (0.224, 0.713)* |  |
| WBC count ($\times$10^9^/L) | 1.003/1.003 | (0.998, 1.009) | | NA | |  |
| **Insulin resistance** | | | | | | |
| LD/CRT^a^ | 1.284/1.163 | (0.475, 2.841) | | 1.694/1.552 | (0.680, 3.549) |  |
| HD/CRT^a^ | 1.220/1.085 | (0.407, 2.777) | | 1.845/1.672 | (0.685, 4.014) |  |
| Age at diag. (yrs) | 0.993/0.994 | (0.910, 1.075) | | 0.989/0.990 | (0.910, 1.070) |  |
| Time since diag. (yrs) | 1.067/1.066 | (0.989, 1.149) | | 1.052/1.051 | (0.980, 1.132) |  |
| Male | 0.547/0.515 | (0.251, 1.031) | | 0.564/0.532 | (0.263, 1.066) |  |
| WBC count ($\times$10^9^/L) | 1.008/1.008 | (1.002, 1.013)* | | NA | |  |
| **(Pre-)hypertension** | | | | | | |
| LD/CRT^a^ | 1.899/1.672 | (0.633, 4.512) | | 1.774/1.565 | (0.607, 4.160) |  |
| HD/CRT^a^ | 2.245/1.840 | (0.555, 6.218) | | 2.000/1.695 | (0.544, 5.246) |  |
| Age at diag. (yrs) | 1.015/1.015 | (0.925, 1.107) | | 1.011/1.010 | (0.924, 1.101) |  |
| Time since diag. (yrs) | 0.978/0.977 | (0.885, 1.074) | | 0.977/0.977 | (0.889, 1.072) |  |
| Male | 4.979/4.367 | (1.817, 11.83)* | | 4.890/4.301 | (1.814, 11.27)* |  |
| WBC count ($\times$10^9^/L) | 0.996/0.997 | (0.986, 1.004) | | NA | |  |
| **Dyslipidemia** | | | | | | |
| LD/CRT^a^ | 2.105/1.978 | (1.021, 3.878)* | | 1.819/1.719 | (0.906, 3.307) |  |
| HD/CRT^a^ | 1.953/1.797 | (0.806, 3.995) | | 1.551/1.442 | (0.686, 3.037) |  |
| Age at diag. (yrs) | 1.037/1.036 | (0.973, 1.102) | | 1.037/1.037 | (0.975, 1.103) |  |
| Time since diag. (yrs) | 1.087/1.087 | (1.023, 1.157)* | | 1.092/1.091 | (1.029, 1.159)* |  |
| Male | 0.986/0.952 | (0.561, 1.613) | | 0.980/0.945 | (0.541, 1.613) |  |
| WBC count ($\times$10^9^/L) | 0.995/0.996 | (0.989, 1.001) | | NA | |  |

LD: low dose corticosteroids; HD: high dose corticosteroids; CRT: cranial radiotherapy; WBC: white blood cell. ^a^: The baseline treatment level is LD/No CRT. ^*^: Credible interval does not contain reference value (significant results).
